# *Photorhabdus *adhesion modification protein (Pam) binds extracellular polysaccharide and alters bacterial attachment

**DOI:** 10.1186/1471-2180-10-141

**Published:** 2010-05-12

**Authors:** Robert T Jones, Maria Sanchez-Contreras, Isabella Vlisidou, Matthew R Amos, Guowei Yang, Xavier Muñoz-Berbel, Abhishek Upadhyay, Ursula J Potter, Susan A Joyce, Todd A Ciche, A Toby A Jenkins, Stefan Bagby, Richard H ffrench-Constant, Nicholas R Waterfield

**Affiliations:** 1 Department of Biology and Biochemistry, University of Bath, Claverton Down, Bath, BA2 7AY, UK; 2 Department of Chemistry, University of Bath, Claverton Down, Bath, BA2 7AY, UK; 3 Centre for Electron Optical Studies, University of Bath, Claverton Down, Bath, BA2 7AY, UK; 4 Department of Microbiology and Molecular Genetics, Michigan State University, East Lansing, Michigan, 48824, USA; 5 School of Biosciences, University of Exeter in Cornwall, Penryn, TR10 9EZ, UK; 6 Department of Microbiology, University College Cork, Cork, Republic of Ireland

## Abstract

**Background:**

*Photorhabdus *are Gram-negative nematode-symbiotic and insect-pathogenic bacteria. The species *Photorhabdus asymbiotica *is able to infect humans as well as insects. We investigated the secreted proteome of a clinical isolate of *P. asymbiotica *at different temperatures in order to identify proteins relevant to the infection of the two different hosts.

**Results:**

A comparison of the proteins secreted by a clinical isolate of *P. asymbiotica *at simulated insect (28°C) and human (37°C) temperatures led to the identification of a small and highly abundant protein, designated Pam, that is only secreted at the lower temperature. The *pam *gene is present in all *Photorhabdus *strains tested and shows a high level of conservation across the whole genus, suggesting it is both ancestral to the genus and probably important to the biology of the bacterium. The Pam protein shows limited sequence similarity to the 13.6 kDa component of a binary toxin of *Bacillus thuringiensis*. Nevertheless, injection or feeding of heterologously produced Pam showed no insecticidal activity to either *Galleria mellonella *or *Manduca sexta *larvae. In bacterial colonies, Pam is associated with an extracellular polysaccharide (EPS)-like matrix, and modifies the ability of wild-type cells to attach to an artificial surface. Interestingly, Surface Plasmon Resonance (SPR) binding studies revealed that the Pam protein itself has adhesive properties. Although Pam is produced throughout insect infection, genetic knockout does not affect either insect virulence or the ability of *P. luminescens *to form a symbiotic association with its host nematode, *Heterorhabditis bacteriophora*.

**Conclusions:**

We studied a highly abundant protein, Pam, which is secreted in a temperature-dependent manner in *P. asymbiotica*. Our findings indicate that Pam plays an important role in enhancing surface attachment in insect blood. Its association with exopolysaccharide suggests it may exert its effect through mediation of EPS properties. Despite its abundance and conservation in the genus, we find no evidence for a role of Pam in either virulence or symbiosis.

## Background

*Photorhabdus *bacteria are pathogens of insects, and obligate symbionts with insect-pathogenic *Heterorhabditid *nematodes [1,2]. These host nematodes invade an insect and regurgitate the bacteria from their gut [3]. The bacteria then colonize the infected insect and release both insecticides that kill the insect host and antibiotics to kill any invading and competing microbes [4]. Following several rounds of nematode and bacterial replication, a new generation of infective juvenile (IJ) nematodes re-uptake the bacteria and exit the cadaver to find new hosts [1]. This dual requirement for symbiosis and virulence makes *Photorhabdus *an excellent model organism for studying bacterial colonization and developmental behaviour in addition to a potential source of potent new insecticidal proteins and antibiotics [2]. The genus *Photorhabdus *comprises three distinct species: *P. temperata, P. luminescens *and *P. asymbiotica*. Although all three are highly pathogenic to insects, *P. asymbiotica *was originally isolated from human wounds and its nematode vector has only recently been identified [5]. Little is known about transmission into human patients, but *P. asymbiotica *is unique in the genus in being able to grow at 37°C and is considered an emerging human pathogen [6]. In an attempt to find potential host-interacting proteins that are relevant to either human or insect infections we used two-dimensional (2D) gel electrophoresis to compare supernatant proteins secreted at 28°C and 37°C. We identified a number of proteins that were differentially produced at these temperatures. Two small proteins were of particular interest, because they were secreted at a very high level at 28°C but were not detectable at the clinically relevant temperature of 37°C. One of these proteins was encoded by a gene on a plasmid found only in *P. asymbiotica *strains. The other was encoded by a chromosomal gene previously identified in a proteomic study of *P. luminescens *TT01 [7]. We present here the first detailed investigation into the role of this second highly secreted protein present in both *P. luminescens *and *P. asymbiotica*.

## Results

### Identification of Pam by two-dimensional electrophoretic analysis of the *P. asymbiotica *ATCC43949 secreted proteins

**Figure 1:**
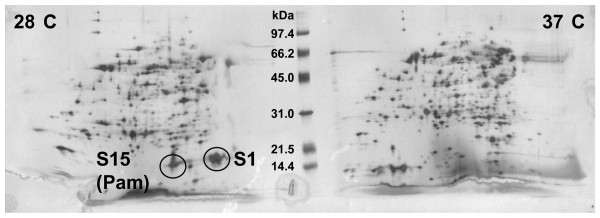
**Two-dimensional gel electrophoresis of the secreted proteome of *P. asymbiotica *ATCC43949**. Proteins were obtained from supernatants of cultures grown at 28°C and 37°C and separated first by isoelectric point, along a 3-10 pH gradient, and then by mass. The 2D gel identifies several proteins with differential levels of production in these conditions, including S1 and S15 (circled) which are only secreted at 28°C.

Given the availability of *P. asymbiotica *ATCC43949 genomic sequence and the ability of this strain to grow at both clinically relevant (37°C) and insect relevant (28°C) temperatures, we used proteomics to identify secreted proteins that may be important for the two different hosts. Two-dimensional gel electrophoresis of supernatant proteins revealed two small highly abundant proteins (initially designated S1 and S15) that were secreted at 28°C but not at 37°C (Fig. 1). We compared the MALDI-ToF profiles of these proteins with a database of all the predicted proteins from the finished *P. asymbiotica *genome sequencing project [8] for their identification. One of these proteins, S1, was found to be encoded by a gene present on the plasmids of clinical *P. asymbiotica *strains but absent from all *P. temperata *and *P. luminescens *strains so far examined. This plasmid, pPAU1, has homology to the *Yersinia pestis *pMT1 plasmid, which is essential for vectoring by the flea host. The small S1 protein is similar to the YPMT1.14c hypothetical protein which has a bacterial Ig-like domain (group 2) although its function is not known. The second protein, S15 (renamed Pam: *Photorhabdus *adhesion modification protein), matched Plu1537 previously identified in proteomic studies of *P. luminescens *TT01 [7]. In strain TT01, the product of the *plu1537 *gene is the most highly secreted protein, accounting for more than 30% of the total extracellular proteins. The *P. asymbiotica *ATCC43949 homologue is a protein of 136 amino acids with a predicted mass of 14.98 kDa and a calculated isoelectric point of 4.7. Searches of current protein databases show limited similarity to known proteins. The best sequence match is seen between amino acids 19-121 of Pam which show 31% identity to amino acids 10-111 of the 13.6 kDa component of a *Bacillus thuringiensis *binary toxin [9]. Injectable insecticidal activity has been reported for Pit, a protein encoded by the homologous gene of *pam *in *P. luminescens *subsp. *akhurstii *strain YNd185 [10]. We used PCR to elucidate the distribution of the *s1 *and *pam *genes in the genus *Photorhabdus *(data not shown). As predicted, the gene encoding S1 was only seen in the plasmid-carrying *P. asymbiotica *isolates and is presumably of relevance only to these strains [8]. An alignment of *pam *sequences from *P. asymbiotica *ATCC43949 and *P. luminescens *TT01 revealed a high level of DNA homology (87.5%). We amplified and sequenced *pam *from 13 other strains of the genus *Photorhabdus*. Sequence comparison of the predicted proteins revealed very high amino acid conservation, with 89.6% similarity between even the most diverse sequences. In addition, the inferred phylogeny of the *pam *genes from different members of the genus follows the same clade-groupings as multi-locus sequence typing data [5] suggesting that *pam *is ancestral to the genus. In order to facilitate further analysis of the Pam protein an antibody was raised to a peptide (KLIQDSIRLDQGEW) conserved in the Pam protein family.

### *In vivo *and *in vitro *production of Pam

**Figure 2:**
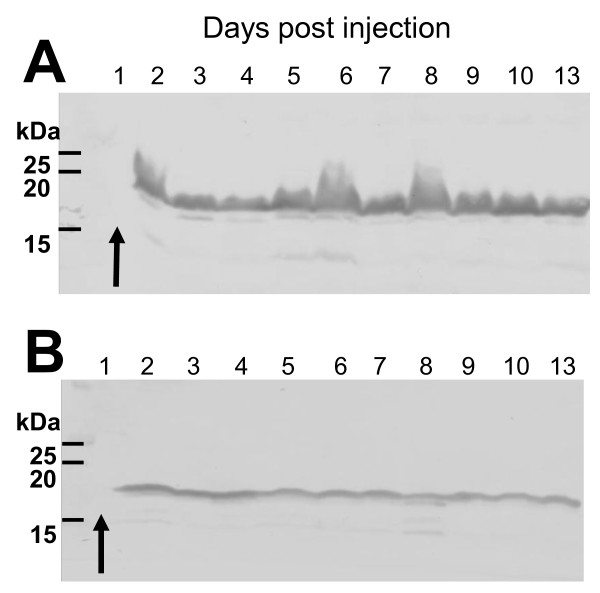
**Detection of Pam in infected *G. mellonella***. Each insect was injected with (A) *P. luminescens *TT01 or (B) *P. asymbiotica *ATCC43949, and was frozen and crushed in 1 ml of buffer at days 1 to 10 and 13 post injection. 10 μl of each sample was used per lane for SDS-PAGE, and Western blot analysis using anti-Pam antibody showed production from the second day after infection. The arrow indicates that Pam is not produced by *Photorhabdus *in the first day of *G. mellonella *infection or that it is below the detection limit of the assay.

**Figure 3:**
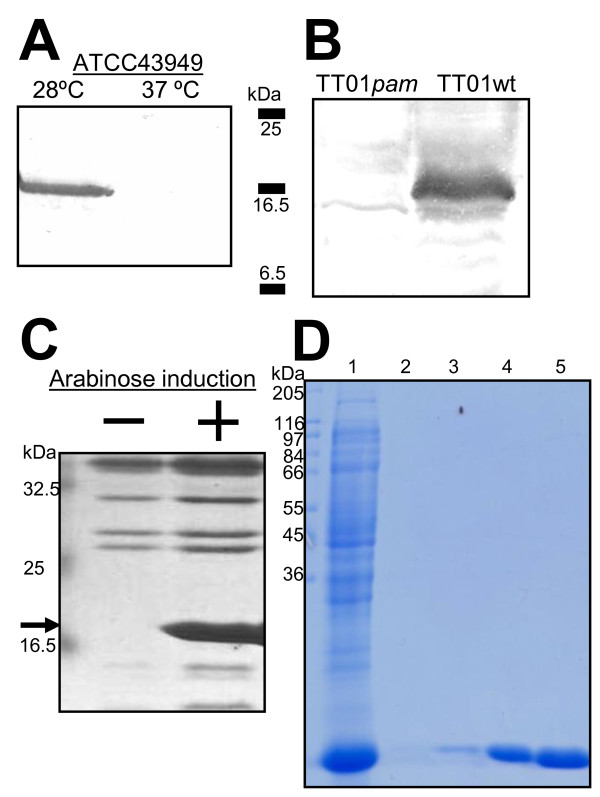
***In vitro *Pam production**. (A) Western blot confirmation of the temperature-dependent secretion of Pam in *P. asymbiotica *ATCC43949: the protein is present in supernatants from cultures grown at 28°C but not at 37°C. (B) Western blot confirmation of the *pam *knock-out in *P. luminescens *TT01 (left) compared with the wild-type strain (right). Note that figures A and B share the same molecular markers. (C) Pam heterologous production in *E. coli*. The arrow shows high levels of the recombinant Pam protein from *P. asymbiotica *ATCC43949 produced in *E. coli*. (D) Pam was purified by two steps of anion-exchange chromatography and the eluted fractions were analysed by SDS-PAGE. Lane 1: Proteins from overnight culture, lanes 2-5: elution fractions from the second ion-exchange column. The estimated purity of recombinant Pam was 95%.

As the identification of highly-secreted Pam occurred at 28°C, a temperature relevant to the infection of insect hosts, we monitored Pam production over time in *Galleria mellonella *larvae injected with either *P. luminescens *TT01 (Fig. 2A) or *P. asymbiotica *ATCC43949 (Fig. 2B). We observed high levels of production in the insect host at 48 h post-injection which continued for a further 11 days, suggesting a possible role of this secreted protein in the occupation of the insect cadaver. It is also possible that Pam is produced in the insect before 48 h and has not been detected with our methods. We were unable to isolate tissues within the insect for Pam-specific production patterns due to internal disruption of the cadaver 48 h after infection. *In vitro *production of Pam was monitored in *P. asymbiotica *ATCC43949 liquid cultures, and it was first detected in supernatants by Western blot after 6 h 30 min of growth in LB medium at 28°C, corresponding to the exponential phase of the culture (Fig. 3A). Pam continued to be produced throughout growth into stationary phase (48 h) and up to 6 day-old cultures (data not shown). As expected, no Pam was released at 37°C although cell-associated Pam could be detected, indicating it is synthesized but not released into the surrounding milieu. The fact that Pam protein is released only at insect-relevant temperatures and the difficulties with genetic manipulation and transformation of *P. asymbiotica *strain ATCC43949, led us to make a *pam *knock-out strain in the well-characterized *P. luminescens *TT01. Figure 3B shows a Western blot demonstrating the absence of Pam in the mutant strain TT01*pam*. For heterologous expression in *E. coli*, *pam *was amplified from *P. asymbiotica *ATCC43949 and cloned in the arabinose-inducible vector pBAD30, under translational control of its native Shine-Dalgarno region. Heterologous production of Pam was confirmed by Western blot (Fig. 3C). The recombinant protein was purified using ion-exchange chromatography for further analysis (Fig. 3D).

### Pam does not influence insect virulence or nematode symbiosis

Given Pam's similarity to a part of the *B. thuringiensis *13.6 kDa Cry toxin and the previous insecticidal studies on the homologous *pit *from strain YNd185 [10], we tested Pam for toxicity to insects. First, we compared the virulence of the TT01*pam *strain with the TT01rif parental strain by injection into *G. mellonella *using standard LT_50 _assays, where approx 100 cells from a diluted overnight culture were injected per insect, and 100 insect larvae were used per treatment. No significant delay in insect death of the TT01*pam *strain (LT_50 _= 49.7 h) relative to the TT01rif (LT_50 _= 48.0 h) was observed, indicating that Pam does not play a major role in insect pathogenicity. We also injected *G. mellonella *and *M. sexta *larvae with a range of dilutions from suspensions of sonicated *E. coli *cells producing Pam, but we saw no toxicity (data not shown). Finally, to assess oral toxicity, we fed *M. sexta *neonate larvae with suspensions of sonicated cells producing Pam. We observed no significant differences in larval weight gain after one week (expressed in average grams ± standard error) between *E. coli *expressing *pam *(0.1165 ± 0.005), *E. coli *control carrying the empty vector (0.0952 ± 0.009) and PBS buffer as control (0.1154 ± 0.010), indicating that Pam does not cause oral toxicity or delay in feeding in *M. sexta*. Our data suggest no role of Pam in insect virulence under the conditions tested.

We examined the ability of TT01*pam *to form an effective symbiosis with the host nematode *Heterorhabditis bacteriophora*. We saw no defect in transmission efficiencies (mean ± s.e.) of TT01*pam *(0.954 ± 0.023) when compared to TT01 wild type (0.954 ± 0.025). We also observed no significant differences between nematodes carrying TT01*pam *and those carrying TT01 wild type when we assessed other traits relevant for symbiosis such as: recovery from infective juveniles (IJ stage) to hermaphrodites (adult stage) and development to second generation *in vitro*, repackaging of the bacteria and infection of *G. mellonella *with re-coupled EPN-complex and emergence yield (data not shown). The results from all the aspects investigated suggest that Pam is not essential either for the symbiotic stage of the *Photorhabdus *lifecycle or for pathogenic activity in the insect.

### Pam binds to EPS in the extracellular matrix and modifies cell attachment

**Figure 4:**
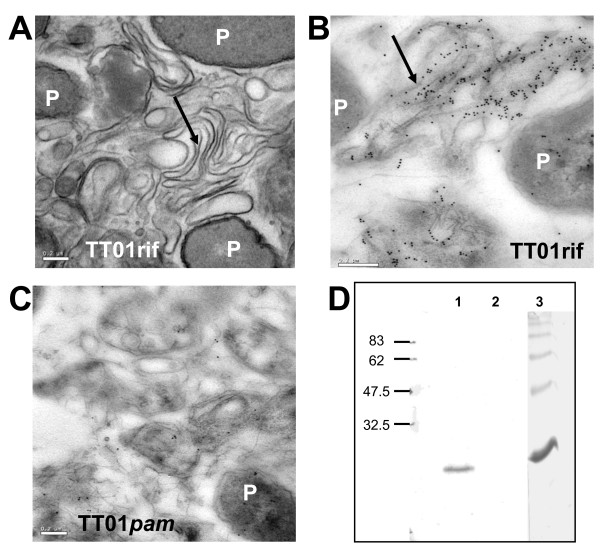
**Pam localization on bacterial cells**. (A) Micrograph of a cross-section from a *P. luminescens *TT01 colony observed by TEM. Note the presence of an extensively folded extracellular matrix (black arrow) between the bacterial cells (indicated with P). (B) Immunolocalization of Pam using the anti-Pam antibody and a conjugated-gold secondary antibody. Gold particles extensively decorate the fibrillar EPS-like matrix (black arrow). (C) The TT01*pam *strain shows no anti-Pam antibody signal but the fibrillar matrix is still present. Scale bars are 0.2 μm. (D) Western blot confirming the presence of Pam in preparations of crude EPS. Lane 1: crude EPS extracted from TT01rif, lane 2: EPS from TT01*pam *and lane 3: purified recombinant Pam.

To investigate the localization of Pam in *P. luminescens *TT01 cells, sections of bacterial colonies were observed under transmission electron microscopy (TEM) revealing large amounts of exopolysaccharide (EPS)-like matrix filling the spaces between cells (Fig. 4A). We used immunogold localization of Pam in these sections and found that the protein is associated with this extracellular material that is distributed surrounding the cells (Fig. 4B). In TT01*pam *the EPS-like material was still present but we did not see specific binding of the antibody (Fig. 4C), suggesting that although Pam binds to the extracellular matrix, it does not significantly alter its production or general structure. Furthermore, Western-blot analysis using the anti-Pam antibody revealed that Pam could be detected in crude EPS preparations (Fig 4D), confirming that from all the extracellular matrix components Pam binds at least to EPS. Our studies revealed that EPS-bound Pam can be released by the action of SDS and salt (KCl) but not by mechanical disruption (vortex) (data not shown).

**Figure 5:**
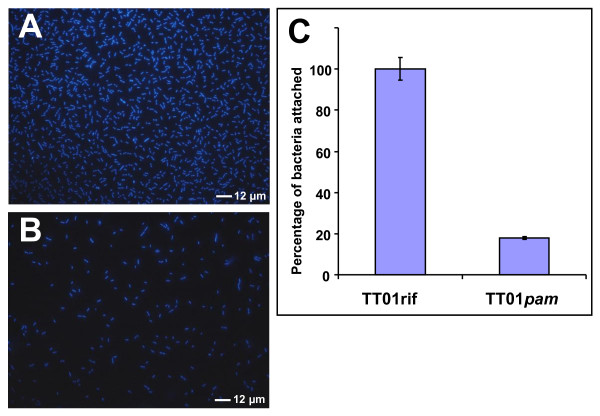
**Comparison of bacterial attachment to surfaces in presence of insect hemolymph by fluorescence microscopy between TTO1rif and the *pam *mutant**. Cells were grown on glass coverslips at 28°C for 8 hours. Planktonic bacteria were washed off and adherent bacteria were fixed and stained with DAPI. The adherence of TT01*pam *(B) is presented as a percentage of the data determined for the corresponding parental strain TT01rif (A). Bacterial counts were performed at 60× magnification and the data represent the mean values of 12 fields from triplicate experiments (± St.Dev) (C).

As Pam binds to EPS and EPS has been shown to be important in biofilm formation [11], we investigated the possibility that Pam influences the different stages of biofilm formation. Pellicle assays and biofilm growth in microscopy chambers did not show differences in mature biofilm formation between TT01rif and TT01*pam *(data not shown). To analyze the influence of Pam on the early steps of biofilm formation, namely initial attachment, we looked at attachment of the two strains to glass coverslips when cultured *ex vitro *in hemolymph plasma. As shown in Figure 5, the parental TT01rif cells attached in greater numbers than TT01*pam *to the glass surface in hemolymph, but not in LB medium or Schneiders insect growth medium (data not shown). Importantly, we were also able to detect Pam in cell and supernatant fractions in bacteria grown in hemolymph plasma at 8 hours.

**Figure 6:**
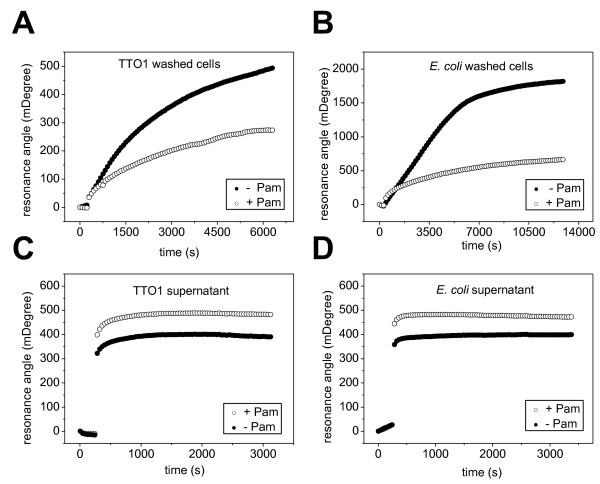
**Surface plasmon resonance analysis of Pam-mediated adhesion on gold-coated glass probes**. (A and B) Presence of the protein on the cell surface (washed cells) showed decreased adhesion to untreated gold surfaces in both TT01rif and *E. coli *pBAD*pam *(+Pam), when compared with the correspondent strains lacking Pam, TT01*pam *and *E. coli *pBAD respectively (-Pam). (C and D) Supernatants from cultures expressing *pam*, TT01rif and *E. coli *pBAD*pam *(+Pam), showed more adhesion than those lacking the protein TT01*pam *and *E. coli *pBAD (-Pam), indicating the ability of free Pam to adhere to surfaces.

To study in more detail the role of Pam in attachment and its adhesive properties, we used surface plasmon resonance (SPR) to measure binding to an abiotic gold surface. First, we used washed cells in an attempt to assess the role of Pam when it is bound to the EPS surrounding the bacterium: TT01*pam *showed increased binding to the surface compared to the parental TT01rif (Fig. 6A), indicating that the presence of the protein reduces adhesion to the surface in these conditions. Similarly, in Pam-expressing *E. coli *we observed a decrease in adhesion compared to *E. coli *control (Fig. 6B). Using SPR to assess the effect of Pam secreted into the medium, we analyzed the supernatants of cultures. In this case we found the opposite effect: when Pam, either from TT01rif or recombinant *E. coli *cultures, was secreted in the supernatant we observed a greater change in SPR angle, indicating that in the presence of Pam more material bound to the gold surface than from the supernatant of cells lacking Pam, TT01*pam *and control *E. coli *(Figs. 6C and 6D). We checked that this effect was due specifically to the presence of Pam in the supernatant by blocking Pam binding with addition of the anti-Pam antibody (X. Muñoz-Berbel, M. Sanchez-Contreras and A. T. A. Jenkins, unpublished data). These results suggest that secreted Pam binds to surfaces, while when Pam is bound to the cell surface it makes these cells less able to attach.

### Structural studies of Pam

**Figure 7:**
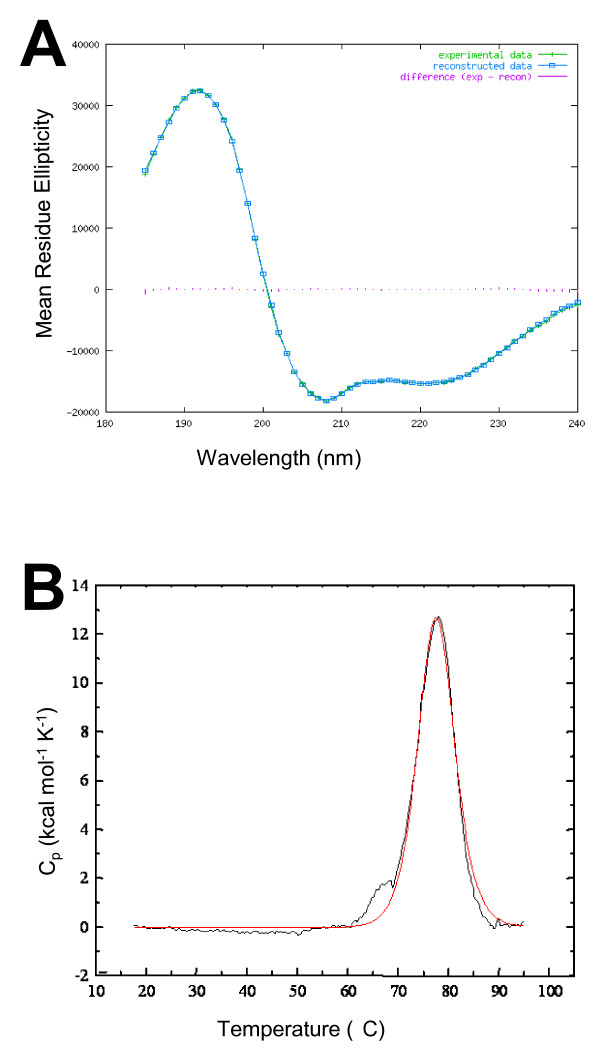
**Structural properties of Pam**. (A) Graphical output of far-UV CD data for Pam reveals that experimental data (green crosses) and calculated spectrum (blue boxes), derived from the calculated output secondary structure, show agreement. The difference spectrum (purple lines) is very close to zero throughout the wavelength range, indicating the goodness of fit of the structural predictions. The CD data indicate that Pam is largely helical (58%), with only a small fraction of residues forming β-strands. (B) Thermal stability of Pam measured by differential scanning calorimetry. The normalised thermal transition curve (red line) shows energy uptake by Pam reached a peak (Tm) at 77.4°C, representing the temperature at which 50% of the protein molecules are unfolded. This was almost identical after cooling the sample and repeating (black line).

In order to better understand the physicochemical properties that confer on Pam the ability to bind EPS and influence cell attachment, we investigated the structural properties of the protein by circular dichroism (CD) spectroscopy and differential scanning calorimetry (DSC). CD spectra at near-UV and far-UV wavelengths were obtained for purified heterologously produced Pam. Weak spectra were recorded in the near-UV, but a strong signal was obtained between 182 nm and 240 nm in the far-UV range. A large positive maximum at 192 nm and negative maxima at 208 nm and 222 nm indicate that the secondary structure of the protein is largely α-helical [12]. Analysis of the spectra using the CDSSTR variable selection method gave secondary structure estimates of 58% helix, 8% strand, 16% turns and 18% unordered structure. The normalized root mean standard deviation (NRMSD) for the estimates provides a goodness-of-fit measure of the correspondence between the experimental and calculated spectra (Fig. 7A); we obtained a NRMSD value of 0.011, which suggests a very accurate prediction of the secondary structure. However, this prediction depends ultimately on how closely the reference dataset proteins used to derive the calculated spectra share structural similarity to Pam [13].

The temperature stability of Pam was measured using DSC. Energy changes in purified recombinant protein were recorded as the sample was heated at a constant rate from 20°C to 95°C. The sample was then allowed to cool before the analysis was repeated. The thermal transition curve measured for Pam reveals two things: firstly, the protein is relatively thermostable, not undergoing a change in enthalpy until the temperature of the system was above 60°C, and reaching a transition midpoint at 77.4°C. Above this midpoint, energy is released and the thermal profile drops toward the baseline (Fig. 7B). Secondly, upon reheating Pam follows a similar profile, except for a slight shoulder between approximately 60°C and 70°C. This shoulder is indicative of misfolding, with the protein not making all of its native contacts, but its magnitude suggests that the protein was largely able to refold to its original conformation and unfold at a rate identical to that measured in the first scan.

## Discussion

We have studied a previously identified protein (Plu1537, here renamed Pam) which in *P. asymbiotica *ATCC43949 is secreted in a temperature-dependent manner, suggestive of a host-specific role in insects. In the closely related insect-only pathogen *P. luminescens *TT01, Pam has been estimated to constitute more than 30% of total secreted protein [7], potentially indicating an important role in the lifestyle of this bacterium. Further sequence alignment and the inferred phylogeny of the *pam *genes from different *Photorhabdus *species suggest that *pam *is both ancestral and conserved throughout the genus. Where variable regions in amino acid sequence do exist, they could therefore be responsible for determining functional specificity of the protein within strains.

Given the characteristic dual lifecycle of *Photorhabdus*, with both a nematode-symbiotic and a insect-pathogenic stage, the limited similarity of Pam with *B. thuringiensis *Cry34 insecticidal protein, and the previous insecticidal studies with Pit [10], the first phenotypes tested with the *pam *mutant were toxicity to insects and symbiotic efficiency with the bacterium's partner nematode *H. bacteriophora*. Interestingly, the deletion of the *pam *gene did not affect the ability of *P. luminescens *TT01 to support nematode growth, the production of infective juveniles, re-association of the bacteria with the worm or their ability to re-infect an insect. Similarly, we were not able to demonstrate any difference in insect survival (measured by LT_50_) when *G. mellonella *were injected with wild-type or *pam *mutant strains, but this could result from the high redundancy of virulence factors in *Photorhabdus *[14]. In the case of Pam recombinant protein, which did not cause toxicity either by injection or feeding assays, it is possible that Pam is not toxic by itself but requires a second, as yet unidentified, protein partner that operates in a binary toxin-type system. The closest known homolog of Pam is the 13.6 kDa Cry34 protein from *B. thuringiensis*, which only exerts effective mortality when coupled with its partner Cry35 [15,16]. The precise mode of action of Cry34 toxins remains unclear, but susceptible insects show histopathological symptoms in the midgut epithelium, characterized by cell blebbing and vacuolation [9]. We have not found any genes in *Photorhabdus *that are predicted to encode a component similar to Cry35. It should be noted that our findings are contrary to reports of toxicity of purified Pam protein by Li and co-workers [10]. It is possible that the Pam variant they produced (Pit) as a GST-fusion from *P. luminescens *subsp. *akhurstii *YNd185, either has a much greater inherent toxicity to *G. mellonella*, or that the different method of purification used by these authors preserved Pam's toxic phenotype.

The fact that we did not find any toxic effect of Pam towards insects, or any decrease in the efficiency of interaction with the symbiotic nematode, led us to investigate whether it was expressed during insect infection at all. Western blots with anti-Pam antibody against proteins isolated from infected insects suggested that Pam was first produced at 48 h and not earlier during the infection process, and that it was continuously produced for at least 11 days after insect death. Although the possibility exists that earlier production was below the detection limit of our assay, we note that 48 h coincides with death of the insect as determined by LT_50 _assays. These results indicate that Pam may play a role in occupancy of the insect cadaver rather than killing of the host and are consistent with a previous study of *P. luminescens *genes upregulated upon insect infection, in which *pam (plu1537) *was not present among the identified genes encoding several toxins and metabolic enzymes [17]. We have detected Pam both as secreted protein in the extracellular medium and bound to the EPS decorating the extracellular matrix surrounding cells. However, the observable structure of EPS/matrix is not significantly altered by the presence or absence of Pam. Although we observed no differences in mature biofilm, we found that Pam influences the early stages of bacterial attachment in hemolymph. SPR data from *E. coli *and *P. luminescens *cultures showed that membrane-bound Pam reduces the ability of cells to bind to the abiotic surface of the metallic gold of the probe, and that the secreted protein itself is able to bind to this surface. The observation that Pam expression increases binding to an abiotic surface in insect blood is in contrast to the findings from the SPR analysis which suggest Pam lowers the adhesive properties of the cell. However these observed differences in attachment between the wild type and *pam *mutant in the hemolymph are not directly comparable with the SPR data. In the first case the cells are grown in the media where attachment is assessed and the combination of secreted and cell-bound Pam contributes to the phenotype, while for SPR we analyzed washed cells and supernatant separately. Furthermore, insect blood is a far more complex environment than the PBS used to resuspend the cells in the SPR study, so potential interactions of Pam and the bacterium with components of the insect immune system must be considered. Together, these data indicate that Pam is a secreted adhesive factor that modifies the surface properties of the cell, affecting the attachment process, specifically cell-to-cell and cell-to-surface attachment. Although it is important to note that attachment to abiotic substrata is not the same as attachment to living or devitalized tissue, we believe that this modification of adhesion by Pam may be involved in one or several processes key to the biology of the bacterium. For instance, once *Photorhabdus *has been regurgitated by IJ nematodes, it must colonize and invade the midgut [4] and this establishment of a biofilm, following attachment, is recognized as an important step in many microbial infections [18]. Since the effect of deleting Pam does not result in a complete gain or loss of attachment, the protein may allow some plasticity in colonization during the infection. Perhaps Pam allows adhesion to specific tissues or a transient attachment, or even to facilitate the release of cells from biofilms to colonize other tissues within the host, acting in an analogous manner to glycanases described other biofilm-forming bacteria [19].

The predicted amino acid sequence of Pam gives little clue to its role or of the potential structure that mediates its adhesive properties. To get an insight into the structure of Pam, we analyzed the protein with circular dichroism spectroscopy. Our far-UV CD data strongly indicate that Pam is a helical protein, with 5.5 helix segments per 100 residues and an average helix length of 10.5 residues. By contrast, only 8% of residues are expected to form β-strands. We obtained only very weak spectra for Pam in the near-UV wavelengths, but 1D ^1^H and 2D ^1^H-^15^N HSQC NMR spectra (data not shown) and high melting temperature from differential scanning calorimetry experiments confirm that the protein has well defined tertiary structure. A degree of tertiary structural prediction is available from the far-UV spectra, specifically the position of the spectral cross-over from positive to negative, and the magnitude of the negative maximum at 208 nm [20]. These both suggest that Pam is a α+β protein. Rather than having intermixed segments, such proteins have separate α-helix and β-sheet-rich regions [21]. Interestingly, although Pam is not secreted at 37°C in *P. asymbiotica*, it shows thermal stability far beyond this. Differential scanning calorimetry revealed that the protein does not begin to thermally denature until heated to temperatures above 60°C. The transition midpoint is 77.4°C, suggesting that Pam is particularly thermostable for a protein produced by an organism considered to be psychrophilic [22]. In fact, this midpoint is approaching that seen in thermophilic bacteria and archaea [23-25]. Without high resolution structural analyses we are unable to explore precise contributions to the thermal stability of Pam, but the high α-helix content is likely to be significant; thermostable proteins are richer in α-helices than mesophilic proteins [26]. The observed ability of Pam to refold to its native conformation following denaturation may be biologically significant; this folding indicates that the protein can form its native structure in the absence of molecular chaperones, outside of the cell if it is secreted as an unfolded polypeptide. It is as yet not clear how Pam is secreted from the cell as we can detect no recognizable signal motifs, neither were found in Pit [10].

Finally, although the role of this highly secreted protein in *Photorhabdus *biology has not yet been completely elucidated, we have shown its possible relevance in cell attachment. Our findings indicate that Pam is a secreted adhesive factor of *Photorhabdus *that modifies attachment of cells to surfaces in biotic (hemolymp) and abiotic (SPR) conditions. Thus, it might be involved at different cell-cell and cell-surface adhesion stages during the insect host colonization, such as the production of a biofilm-like matrix on the insect gut, the spread of bacteria within the insect cadaver and potentially a resource protection role, binding to the insect tissues and preventing other saprophites from taking advantage of the biomass. The high levels of secretion and the degree of conservation within the genus are congruent with Pam modulating these important activities. Very little is known about *Photorhabdus *infections in humans, but a recent study has found that, unlike the extracellular growth of *P. luminescens *in insects [27], a clinical isolate of *P. asymbiotica *is a facultative intracellular pathogen when incubated with human macrophage-like cells [28]. Future studies may investigate what role if any Pam has in the infection of mammalian cells.

## Conclusions

In this study we show that the highly abundant Pam protein is able to bind to exopolysaccharides and change the attachment properties of *Photorhabdus*. Deletion of *pam *altered bacterial adhesion to surfaces but did not cause a decrease in virulence towards *Galleria mellonella *larvae. However, Pam is produced during insect infection suggesting a role for this protein in the insect cadaver, possibly in the colonization of the insect body. Sequence analysis of *pam *in multiple isolates showed that it is ancestral and conserved in the genus *Photorhabdus *and thus deserves further investigations to clarify its role in the complex cycle of *Photorhabdus *biology.

## Methods

### Bacterial strains, plasmids and culture conditions. DNA amplification and cloning

The strains used in this study are: *P. asymbiotica *strain ATCC43949 [29], *P. luminescens *subspecies *laumondii *strain TT01 [30] and a wild-type spontaneous rifampicin-resistant *P. luminescens *TT01rif (this study). A knock-out strain in the *pam *gene was constructed from TT01rif and named TT01*pam*. The *pam *gene was deleted from the chromosome by allelic exchange using the suicide vector pDS132 [31] and correct chromosomal deletion was confirmed by PCR and DNA sequencing of the region near the deleted gene. The *pam *knock-out strain grew similarly to the wild-type strain in rich and minimal media and insect plasma (filtered hemolymph). *Escherichia coli *EC100 (Epicentre Biotechnology, USA) was used for heterologous production of Pam. The *pam *gene was PCR amplified from *P. asymbiotica *ATCC43949 genomic DNA using the primers PamF: 5' TTAATCTTGGAATTCATTAAACACATT 3' and PamR: 5' TTAAAGCTTAGGTTACAATAGTATATTCT 3'. Using *Eco*RI and *Hin*DIII restriction sites incorporated in the primers, the product was directionally cloned downstream of an arabinose-inducible promoter in the pBAD30 plasmid [32] to create the pBAD*pam *expression construct. Pam expression in *E. coli *EC100 containing pBAD*pam *was induced by addition of 0.2% (w/v) L-arabinose overnight, and *E. coli *EC100 carrying pBAD30 empty vector was used as control. Cloned *P. asymbiotica *ATCC43949 *pam *in pET-28α (Novagen, USA) and expressed in *E. coli *BL21 (DE3) (Novagen, USA) was used for the feeding assays, and compared to *E. coli *EC100 carrying pET-28α as control. Strains were grown with 250 rpm agitation on LB medium [33] supplemented when required with 50 μg ml^-1 ^rifampicin, 100 μg ml^-1 ^ampicillin, 10 μg ml^-1 ^tetracycline and 100 μg ml^-1 ^kanamycin. *E. coli *strains were grown at 37°C, *P. luminescens *TT01 and its derivatives at 28°C and *P. asymbiotica *at both temperatures depending on the assay. For pellicle assays [34] and biofilm in microscopy chambers (Ibidi) strains were grown statically in LB and Grace's/Schneider's insect media (Sigma).

Amplification of the *s1 *gene from *P. asymbiotica *isolates was performed using the primers s1F: 5'TATGAATTCATAAGTAAGGAT 3' and s1R: 5' CGGTGTTTTAGTAAGCTTCTATCT 3'.

### Two-dimensional gel electrophoresis, Western blot and Pam protein purification

From a starting overnight culture (28°C) of *P. asymbiotica *ATCC43949, cultures were inoculated and grown for 24 h at 28°C and 37°C until early stationary phase. Proteins from both supernatants were phenol precipitated and resuspended in 150 μl CDU buffer (4% CHAPS, 130 mM DTT and 9 M Urea) containing 1 × HALT^TM ^protease Inhibitor Cocktail Mix (Pierce, Thermo Fisher, UK). Samples were incubated for 2 h at room temperature, then centrifuged for 30 min at 88 760 × g. The RediPlate Protein Quantitation Kit (Molecular Probes, Invitrogen, UK) was used to quantify protein concentration in the samples and equivalent amounts of total proteins were loaded. A Multiphor II system (GE Healthcare, UK) was used for isoelectric focusing and horizontal SDS-polyacrylamide gel electrophoresis with Immobiline DryStrip gels and precast 12.5% SDS gels (GE Healthcare, UK), following the manufacturer's instructions. Gels were Coomassie stained and protein spots were excised and sent to the protein sequencing facility at the University of the West of England (Bristol, UK). The peptide sequences resulting from MALDI analysis of trypsin-digested proteins, were compared to all proteins in the SwissProt non-redundant database and to a database of predicted proteins from the *P. asymbiotica *ATCC43949 genome sequence [8].

A polyclonal anti-Pam antibody was raised in rabbits against the peptide KLIQDSIRLDQGEW (amino acid positions 28-41) from *P. asymbiotica *ATCC43949 by GenScript Corporation (USA). For Western blot, proteins were precipitated with 1/10 volumes of 100% Trichloroacetic acid, separated by SDS-PAGE and transferred onto a Trans-Blot nitrocellulose membrane (BioRad, USA) using a Semi-Dry blotter (BioRad, USA). Membranes were incubated with 1/500 dilution of the anti-Pam antibody for 90 min and with 1/5000 dilution of an anti-rabbit alkaline phosphatase conjugated secondary antibody for 90 min Alkaline phosphatase reaction with NBT-BCIP solution (Fluka, Sigma-Aldrich, USA) was used for development. To detect production of Pam *in vivo*, larvae of *Galleria mellonella *were injected with 20 μl of diluted overnight cultures of either *P. luminescens *TT01 or *P. asymbiotica *ATCC43949, corresponding to 200 CFU. Infected insects were collected on successive days and crushed in lysis buffer, containing 125 mM Tris pH 8.0, 4 M urea, 2% SDS, and 5% β-mercaptoethanol (1 ml per insect). From each sample 10 μl were loaded for SDS-PAGE and analyzed by Western blotting.

For the purification of recombinant Pam: The pellet of 1 liter of *E. coli *cells producing Pam was resuspended in 10 ml of buffer A (20 mM HEPES pH 7.5, 50 mM NaCl) and lysed by sonication. The insoluble fraction was pelleted by centrifugation at 4°C, 16 000× g, 20 min and the resulting supernatant was diluted to 20 ml with buffer A. This supernatant was loaded as 5 ml fractions onto a 5 ml Hitrap QFF anion exchange chromatography column (GE Healthcare, UK) equilibrated with: 3 × column volumes (cv) buffer A, 3 × cv buffer B (20 mM HEPES pH 7.5, 1 M NaCl) and 3 × cv buffer A. Chromatography was performed on an ÄKTA purifier (GE Healthcare, UK). The column was run at 0.8 ml min^-1 ^with a 15 ml wash after loading and a 5 × cv gradient from 5% to 100% buffer B to elute the protein. 1 ml fractions were collected and 10 μl samples were loaded for SDS-polyacrylamide gel electrophoresis. The Hitrap QFF step was followed by further anion exchange using a 1 ml MonoQ column (GE Healthcare, UK). Fractions containing Pam were diluted fourfold with buffer A and 4 ml were loaded after equilibration of the column. Pam was eluted with a gradient of 5%-25% buffer B over 8 cv, and fractions containing Pam were identified by SDS-PAGE. The estimated purity of Pam was 95%.

### Extracellular-polysaccharide (EPS) crude extraction

Cells grown on LB agar were harvested with a minimal volume of 0.9% NaCl solution and EPS was detached by mixing for 15-20 s in a blender. Cells were pelleted and discarded, and 3 volumes of chilled acetone were added to the EPS-containing supernatant (previously concentrated to 30-40 ml by freeze-drying). The mixture was kept at -20°C overnight, centrifuged at 3 000 × g for 20 min and the pellet was dried and resuspended in a small volume (10-20 ml H_2_O). This sample was ultra-centrifuged at 100 000 × g for 4 h to precipitate the lipopolysaccharide fraction. The supernatant was removed and dialyzed overnight at 4°CC. Samples were frozen at -80°C for 4-6 h, and freeze-dried to concentrate. EPS suspensions (2 mg/ml) from TT01rif and TT01*pam *were analysed by SDS-PAGE and Pam was detected by Western blot. A suspension of TT01rif EPS (5 mg/ml) was incubated with 1.6% SDS or salt (0.5 M KCl) or vortex for 4 mins before performing electrophoresis on native gel and Western blot.

### Virulence, toxicity and symbiosis assays

For calculation of the LT_50_, or time taken for half of the initial population to die, approx 100 cells from overnight cultures of either TT01rif or TT01*pam *were injected per insect and 100 *G. mellonella *larvae were used per treatment. LT_50 _is the calculated time after injection at which 50% of the larval population was dead; differences in LT_50 _times represent different rates of killing. Scoring of insect death was carried out every 2 h between 44-52 h and 59-68 h post-injection. Oral toxicity was assessed with a standard feeding bioassay: 1 cm^3 ^artificial wheat germ food blocks were supplemented with 200 μl of supernatant from sonicated and pelleted *E. coli *cultures or Phosphate Buffered Saline (PBS) as control. All supplements were allowed to dry for 20 min under laminar flow. Two first-instar *Manduca sexta *neonate larvae were placed on each food block (12 larvae per treatment) and after 7 days incubation at 25°C the weight of each caterpillar was recorded. Tobacco hornworm *M. sexta *larvae were maintained on a wheat germ-based artificial diet [35] at 25°C with a photoperiod of 17 h light: 7 h dark. *G. mellonella *larvae were supplied by Livefood (UK).

For the assessment of symbiosis, infective juvenile (IJ) *H. bacteriophora *were propagated on a lawn of either TT01rif or TT01*pam *cells constitutively producing GFP. The proportion of IJs transmitting GFP-labeled bacteria was determined by fluorescence microscopy, as previously described [36].

### Transmission electron microscopy and hemolymph attachment assays

For transmission electron microscopy (TEM), bacterial colonies grown on LB plates were fixed in 2.5% glutaraldehyde for 2 h, washed in PBS and post-fixed in 1% osmium tetroxide for 1 h. Samples were dehydrated in acetone and embedded in Spurr's resin (TAAB, Premix). Sections were cut with an ultra-microtome (Leica, Reichert Ultracut E) and stained with uranyl acetate and lead citrate before examination with a JEOL JEM 1200 EXII transmission electron microscope (JEOL Tokyo, Japan). For labeling Pam at the ultra-structural level, samples were dehydrated in ethanol and embedded in LR White acrylic resin before sectioning and incubation with the primary (anti-Pam) and secondary antibodies (goat anti-rabbit IgG conjugated with 10 nm gold particles).

For analysis of attachment in 5^th ^instar *M. sexta *plasma (filtered hemolymph), overnight cultures of TT01rif wild-type and TT01*pam *were diluted to 0.05 OD_600 _and incubated at 28°C for 8 h without agitation in 24-well tissue culture plates on sterile circular glass coverslips. After 8 h the planktonic bacteria were gently aspirated and the coverslips were washed 3 × with PBS. The coverslips were fixed with 300 μl of 4% PBS-paraformaldehyde at 4°C for 24 h and stained with 4',6-diamidino-2-phenylindole (DAPI). Coverslips were mounted with Miowiol (Calbiochem) and analyzed with fluorescent microscopy (Nikon Eclipse 90i, Japan). Cells count at 8 h was the average in 12 fields of vision at 60× magnification from triplicate samples for the TT01rif and the same sample size for the TT01*pam *mutant.

### Physicochemical methods

Surface plasmon resonance (SPR) data were acquired using the Autolab ESPRIT (Eco Chemie, BV, The Netherlands). Measurements were made by following the variation in the reflected light minimum angle with time, which is indicative of the change in optical properties of the interface as bacteria attach. Glass disks with a thin gold coating (50 nm thick) were placed on a hemispherical prism using index match fluid (RI = 1.518, Cargille), which ensured optical continuity. 50 μl of PBS were added and the system was allowed to stabilise for four minutes before addition of 50 μl of bacteria washed with PBS and adjusted to 6 × 10^8 ^CFU ml^-1^. The minimum angle was thus recorded with time. Measurements were made every three seconds for the duration of the experiment (until the SPR readings stabilized).

Purified Pam at 1 mg ml^-1 ^concentration in 5 mM phosphate buffer, pH 6.0 was used for circular dichroism (CD) spectroscopy and thermal analysis (differential scanning calorimetry, DSC). CD spectroscopy was performed by Sharon Kelly at the Department of Chemistry, University of Glasgow (UK). For CD in far-UV wavelengths, the sample was diluted to 0.383 mg ml^-1 ^and data were collected from a 0.02 cm pathlength cuvette. For CD spectroscopy in the near-UV range, a 0.5 cm pathlength cuvette was used and Pam was diluted to 0.772 mg ml^-1^. The CD spectra, obtained below 550 V, were analyzed using the CDSSTR variable selection method at the Dichroweb server [37,38]. Reference spectra set 3 [39], covering wavelengths 240-185 nm, gave the most consistent results when the analysis was iterated. DSC was performed on a Microcal VP-DSC spectrometer at the BBSRC Microcalorimetry Service (Department of Chemistry, University of Glasgow, UK).

## Authors' contributions

RTJ, MSC and IV carried out experiments and drafted the manuscript. MRA, GY and AU performed experiments and interpreted data. XMB, ATAJ and SB carried out the physicochemical experiments and interpreted data. UJP, SAJ and TAC participated in the acquisition, analysis and interpretation of data. RHffC and NRW obtained funding for and designed the research and critically revised the manuscript. All authors read and approved the final manuscript.
